# Telomere Shortening in the Esophagus of Japanese Alcoholics: Relationships with Chromoendoscopic Findings, ALDH2 and ADH1B Genotypes and Smoking History

**DOI:** 10.1371/journal.pone.0063860

**Published:** 2013-05-07

**Authors:** Junko Aida, Akira Yokoyama, Naotaka Shimomura, Ken-ichi Nakamura, Naoshi Ishikawa, Masanori Terai, Steven Poon, Masaaki Matsuura, Mutsunori Fujiwara, Motoji Sawabe, Tomio Arai, Kaiyo Takubo

**Affiliations:** 1 Research Team for Geriatric Pathology, Tokyo Metropolitan Institute of Gerontology, Tokyo, Japan; 2 Clinical Research Unit, National Hospital Organization Kurihama Medical and Addiction Center, Yokosuka, Kanagawa, Japan; 3 Terry Fox Laboratory, British Columbia Cancer Research Centre, Vancouver, Canada; 4 Department of Cancer Genomics, The Cancer Institute, The Japanese Foundation for Cancer Research, Tokyo, Japan; 5 Department of Pathology, Japanese Red Cross Medical Center, Tokyo, Japan; 6 Department of Moleculo-genetic Sciences, Division of Biomedical Laboratory Sciences, Graduate School of Health Care Sciences, Tokyo Medical and Dental University, Tokyo, Japan; 7 Department of Pathology, Tokyo Metropolitan Geriatric Hospital, Tokyo, Japan; The Chinese University of Hong Kong, Hong Kong

## Abstract

Chromoendoscopy with Lugol iodine staining provides important information on the development of squamous cell carcinoma (SCC). In particular, distinct iodine-unstained lesions (DIULs) larger than 10 mm show a high prevalence in high-grade intraepithelial neoplasia. It has also been reported that inactive ALDH2*1/*2 and less-active ADH1B*1/*1, and smoking, are risk factors for esophageal SCC. We previously examined telomere shortening in the esophageal epithelium of alcoholics, and suggested a high prevalence of chromosomal instability in such individuals. In the present study, we attempted to analyze telomere lengths in 52 DIULs with reference to both their size and multiplicity, ALDH2 and ADH1B genotypes, and smoking history. Patients with DIULs <10 mm (n = 42) had significantly longer telomeres than those with DIULs ≥10 mm (n = 10, *p* = 0.008). No significant differences in telomere length were recognized between the ALDH2 and ADH1B genotypes (ALDH2 active/inactive = 35/17, ADH1B active/inactive = 32/20; *p* = 0.563, 0.784, respectively) or among four groups of patients divided according to smoking history (never-, ex-, light, and heavy smokers = 3, 6, 21, and 22 patients, respectively; *p* = 0.956). Patients without multiple DIULs (n = 17) had significantly longer telomeres than patients with multiple DIULs (n = 35, *p* = 0.040). It is suggested that alcoholism reduces telomere length in the esophagus, irrespective of genotype or smoking habit. Telomere shortening may not generate cancer directly, but may create conditions under which SCC can develop more easily, depending on subsequent exposure to carcinogens.

## Introduction

Squamous cell carcinoma (SCC) is still the predominant histologic type of esophageal cancer in areas where there is a high risk of developing it. Turkish, Mongolian, and African-American populations have a risk of developing esophageal cancer two to three times higher than that of American Caucasians, regardless of sex [1]. Japanese or Chinese populations, and especially alcoholics in both populations, also have a high risk of esophageal SCC, and thus SCC continues to be an important disease of the esophagus.

Telomeres are repetitive G-rich DNA sequences and associated binding proteins found at the ends of linear eukaryotic chromosomes, and appear to play a key role in preventing genomic instability [2], [3]. The progression of telomere shortening with age may lead to genomic instability during the initial stage of tumorigenesis [4]–[7].

Using Southern blotting and quantitative fluorescence in situ hybridization (Q-FISH), we have demonstrated that telomere shortening occurs in almost all human organs and tissues, including esophageal epithelium, with aging [8]–[10], and have confirmed that the annual telomere reduction rate is 60 bp in normal esophageal epithelium [8]. We have also confirmed the telomere length distributions of different cell types in many tissues [10]–[18]. Moreover, telomeres in uninvolved lingual [11] and esophageal [17] epithelium (background) from cases of SCC *in situ* (CIS) have been shown to be significantly shorter than those in age-matched controls. These results have confirmed that our method for telomere measurement is accurate and reproducible.

For early diagnosis of esophageal neoplastic lesions, chromoendoscopy with iodine staining is an important tool [19]. Chromoendoscopy of the normal esophagus demonstrates a diffuse brown coloration. Distinct iodine-unstained lesions (DIULs) with a maximum diameter of ≤5 mm are suggestive of neoplasia, and DIULs of ≤10 mm are indicative of high-grade intraepithelial neoplasia [20]. There are often multiple areas varying in size and shape that are unstained or only weakly reactive with iodine, or which show various intensities of iodine staining [21]. The presence of multiple DIULs and large DIULs has been frequently observed in alcohol-drinkers with synchronous and metachronous multiple SCCs in the esophagus and head and neck, being strongly associated with the ALDH2*1/*2 and ADH1B*1/*1 genotypes [20], [22], [23].

SCC of the esophagus is quite rare in young people [24], but occurs frequently in the elderly and alcoholics [25], [26]. In a previous study, we demonstrated telomere shortening in the esophageal epithelium of alcoholics relative to non-alcoholics, and indicated that alcohol intake shortens telomeres in the esophageal epithelium [18]. It has been reported that the risk factors for esophageal SCC in Japanese drinkers include the inactive ALDH2*1/*2 and less-active ADH1B*1/*1 genotypes, smoking, and frequent drinking of strong alcoholic beverages straight [27]. Although the ALDH2*1/*2 and ADH1B*1/*1 genotypes are very rare among Caucasians, they account for 13.0% of alcoholics in Japan [28].

In the present study, we analyzed the telomere lengths of DIULs, and examined the relationship between DIUL telomere length and DIUL size or multiplicity. Also, we attempted to clarify whether the inactive ALDH2*1/*2 and/or less-active ADH1B*1/*1 genotype, and smoking, are risk factors associated with telomere shortening in alcoholics.

## Materials and Methods

### Ethical Statement

AY was responsible for aquiring informed consent from the patients included in this study, and the participants provided written informed consent to participate in this study. The ethics committees of Kurihama Medical and Addiction Center and Tokyo Metropolitan Institute of Gerontology approved the consent procedure. All specimens used for this study were obtained from Japanese alcoholic patients who underwent upper gastrointestinal endoscopy at Kurihama Medical and Addiction Center. No inclusion criteria other than being a Japanese alcoholic patient were considered for this study. No information on any of the samples studied was able to identify any of the individual patients included.

### 1. Alcoholic subjects

**Figure 1:**
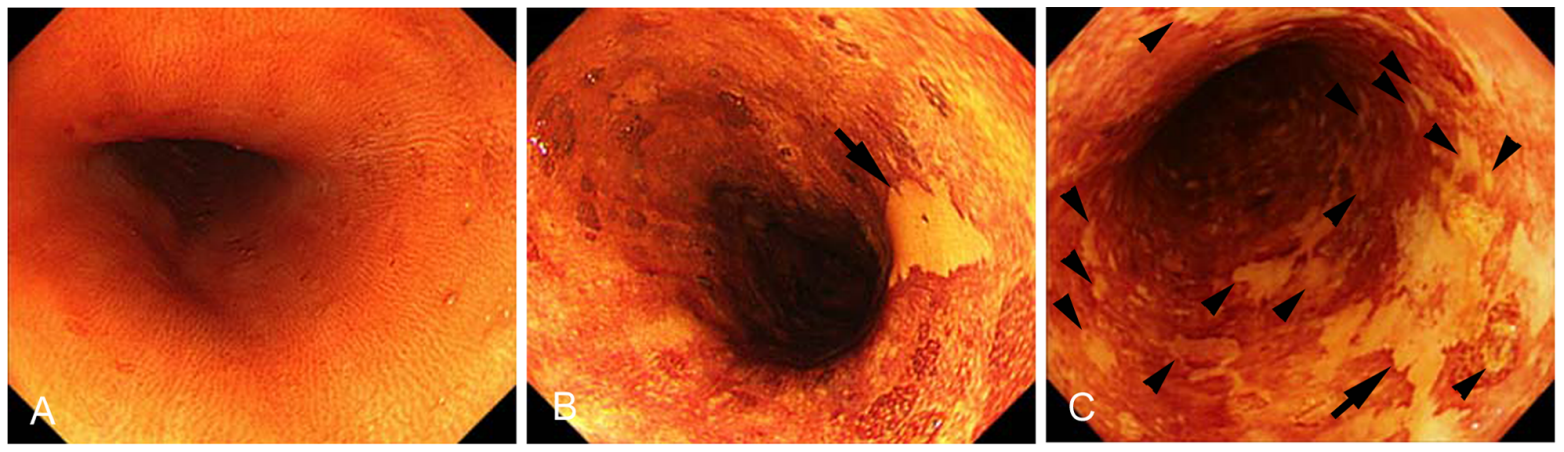
Representative chromoendoscopic images of the esophagus from alcoholics. No distinct iodine-unstained lesion (DIUL) (A), a DIUL (arrow) less than 10 mm in diameter without multiplicity (B), and DIUL over 10 mm in diameter (arrow) with multiple occurrence (arrow head) (C).

Endoscopic examination and biopsy were performed by one (AY) of the authors (2008–2011) at the National Hospital Organization Kurihama Medical and Addiction Center. All of the alcoholics who participated in this study met the DSM-IV criteria for alcohol dependence stipulated by the American Psychiatric Association [29], and underwent screening endoscopy. We examined 96 consecutive alcoholic patients who had no history of head and neck carcinoma (all men, aged 43–82 years, mean: 62.3 years). When a patient was found to have DIULs with a maximum dimension of ≥5 mm, biopsy specimens were taken from each DIUL. A total of 114 such specimens were examined histologically. The sizes of the DIULs from which the biopsy specimens had been taken, and details of the presence or absence of multiple DIULs, were recorded. According to the sizes of the DIULs, we classified the specimens into two groups: DIULs <10 mm, and ≥10 mm. When 10 or more DIULs of any size were observed in one endoscopic field of view, DIULs were recorded as multiple (Fig. 1A–C). Details of smoking history were recorded during the initial visit.

### 2. Tissue processing and histologic assessment

Biopsy specimens were fixed for 5 hours in 10% buffered formalin, and then subjected to standard tissue processing and paraffin embedding. Tissues were sliced serially into sections 3 µm thick for hematoxylin and eosin (HE) staining, and into sections 2 µm thick for Q-FISH.

Histologic examinations of the specimens were performed by 4 pathologists (JA, MS, TA and KT), who were experts in gastrointestinal pathology. Inter-observer variations among histological diagnoses were discussed among these observers using a multi-headed teaching microscope, and a consensus was reached.

Any biopsy specimens with histologically evident atypia, dysplasia, or malignancy were excluded. We used the telomere length of basal cells as the representative value for each specimen, because tissue stem cells are considered to reside in the basal layer [10], [11], [17]. Basal cells were defined as cells in a single layer on the basement membrane [17], [18]. In samples lacking subepithelial tissue, basal cells were defined on the basis of their obvious palisading pattern. Cases lacking a sufficient number of basal cells for analysis were excluded, and 52 specimens without dysplasia were selected for FISH examination.

### 3. ALDH2 and ADH1B genotyping

PCR-restriction fragment length polymorphism was employed to determine the ALDH2 and ADH1B genotypes using DNA samples from blood cells. The samples for this purpose were obtained with informed consent from patients admitted to the National Hospital Organization Kurihama Alcoholism Center, and the data obtained were not released until completion of all the telomere analyses.

### 4. Q-FISH

The slides were processed by the FISH method, as reported previously [10], [12]–[14], [16]–[18].

#### 4.1. FISH and probes

Tissue sections were hybridized with peptide nucleic acid (PNA) probes for the telomere (Telo C-Cy3 probe: 5′-CCCTAACCCTAACCCTAA-3′; catalogue number F1002, Fasmac, Japan) and the centromere (Cenp1-fluorescein isothiocyanate (FITC) probe: 5′-CTTCGTTGGAAACGGGGT-3′; custom-made, Fasmac), and the nuclei were stained with 4′6-diamidino-2-phenylindole (DAPI) (Molecular Probes, Eugene, OR).

#### 4.2. Image analysis of telomeres

FISH digital images were captured by a Charge Coupled Device (CCD) camera (RETIGA-2000DC, QIMAGING, Surrey, BC, Canada) mounted on an epifluorescence microscope (80i, Nikon, Tokyo, Japan) equipped with a triple band-pass filter set for DAPI/FITC/Cy3 (Part #61010, Chroma Technology Corp, Rockingham, VT, USA) and a x40 objective lens (Plan Fluor x40/0.75, Nikon). Microscope control and image acquisition were performed using the Image-Pro Plus software package (version 7.0, Media Cybernetics Co. Ltd., Silver Spring, MD, USA). The captured images were analyzed using our own tissue analysis software, ‘TissueTelo Ver. 3.1’, which estimates the telomere to centromere ratio (TCR) and the normalized TCR (NTCR) of individual nuclei, as reported previously [10], [12]–[14], [16]. Each nuclear region was outlined by hand on the composite color image obtained with DAPI (blue channel), FITC (green) and Cy3 (red) staining. Between 116 and 356 cells (mean: 228.8 cells) in each sample were analyzed. Telomere and centromere signals were then determined as pixels showing the brightest intensities (top 5%) within each selected nuclear region. The measured signal intensities (or optical densities) were corrected for background autofluorescence, as determined from the mean of the pixels showing the lowest intensities (bottom 20%). The top 5% and bottom 20% thresholds had previously been shown to give consistent results [30]. As there is no guarantee that the entire nucleus is captured within any given tissue section, the total corrected telomere signal (the integrated optical density or IOD) for each nucleus was further normalized by the corresponding IOD of the centromere [13], [14]. TCR values were determined from individual cells of the basal layer in samples from alcoholics.

#### 4.3. TCR normalization by cell block

As a control for variations in sample preparation, we also performed Q-FISH on a cell-block section from a cultured cell strain, TIG-1 [31], with a population doubling level (PDL) of 34 (telomere length: 8.6 kbp by Southern blot analysis, kept and cultured at our institution) and placed it on the same slides as the esophageal sections. Each nuclear region in the cell block sections was outlined by hand in the same way as for the basal cells of the esophagus on the composite color image obtained with DAPI (blue channel), FITC (green) and Cy3 (red) staining. Between 114 and 205 cells (mean: 161.4 cells) for each sample were analyzed, and therefore the total number of cells analyzed in each case was 313.0 on average. The mean and median values were calculated and normalized by the median TCR for the control cell block on the same slide to give the mean and median NTCR for the cells [10], [11].

### 5. Chi-square independence test

In order to analyze the independence of chromoendoscopy findings and ALDH2 and ADH1B genotypes, chi-square test was performed among ALDH2 and ADH1B genotypes and the size or multiplicity of DIULs.

### 6. Statistical analyses

The distributions of NTCRs in each case did not show a normal distribution [10], [11], and thus the median NTCR was appropriate for representing the data in each case. However, as the mean value reflects the presence of cells with extremely long telomeres, i.e. tissue stem cells, as well as the median values, we also examined the mean values. The mean and median NTCRs for basal cells were compared between the ALDH2 and ADH1B genotypes, and also the size and number of DIULs. For comparisons of DIUL size and multiplicity, the one-tailed test was applied. With regard to smoking habits, we compared the mean and median NTCRs among 4 groups of patients classified according to smoking history (for details, see Results section) using the Kruskal-Wallis test. For all comparisons, differences at *p*<0.05 were considered to be significant.

## Results

### 1. Analysis of the subjects

#### 1.1. Findings of chromoendoscopy with iodine staining

**Figure 2:**
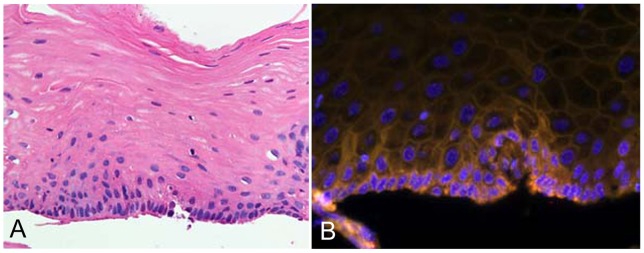
Representative histologic features of the esophageal epithelium of an alcoholic. The biopsy specimen was obtained from an alcoholic 46-year-old man. The specimen was biopsied from a DIUL 15 mm in diameter. HE, x40: There is no evident dysplasia in the epithelium. This subject's mean and median NTCR values in the basal cells were 0.93 and 0.92, respectively (A). FISH image, ×40: Red, telomere-Cy3 signal; green, centromere-FITC signal; blue, DAPI counterstaining for DNA (B).

The 52 cases analyzed were divided into two groups on the basis of the size of the DIUL from which the biopsy specimen had been taken: <10 mm (42 cases) and ≥10 mm (10 cases). Thirty-five of the 52 cases had multiple DIULs and 17 cases did not have multiple DIULs. Finally, 52 cases without dysplasia (Fig. 2A) (patient age 43–82 years, mean 61.5 years) were analyzed by Q-FISH. The histology of mucosal specimens was unrelated to the size or number of DIULs present.

The mean ages of patients whose DIULs measured <10 mm or ≥10 mm, and those of patients with and without multiple DIULs, are shown in Table 1. No significant age differences were evident between the groups.

**Table 1:** Comparison Between Groups of Patients Divided According to ALDH2 and ADH1B Genotypes, DIUL Size, Multiplicity, and Smoking History.

		Number of cases	Mean age ± SD**	Mean NTCR	*p*-value	Median NTCR	*p*-value
ALDH2	*ALDH2* * *1/* * *1* (active)	35	62.9±8.4	1.2581	0.5630	1.1298	0.4499
	*ALDH2* * *1/* * *2* (inactive)	17	58.5±12.0	1.3250		1.2025	
ADH1B	*ADH1B* * *1/* * *2* or * *2/* * *2* (active)	32	61.5±10.1	1.2928	0.7837	1.1639	0.7715
	*ADH1B* * *1/* * *1* (less active)	20	61.4±9.6	1.2595		1.1371	
Diameter of DIULs*	<10 mm	42	61.6±9.6	1.3247	0.0075^#^	1.1844	0.0581
	≤10 mm	10	60.7±11.3	1.0923		1.0242	
Multiple DIULs*	Isolated DIULs	17	60.1±7.4	1.4640	0.0403^#^	1.3119	0.0275^#^
	Multiple DIULs	35	62.1±10.8	1.1906		1.0767	
Smoking history**	Never smoked	3	56.7±13.6	1.1797	0.7543	1.1803	0.9561
	Ex-smoker	6	64.3±7.9	1.2360		1.1249	
	Light smoker	21	58.4±9.5	1.2332		1.1348	
	Heavy smoker	22	64.2±9.6	1.3505		1.1753	

* : one-tailed *t* test.

DIUL: Distinct iodine-unstained lesion.

** : Kruskal-Wallis test.

#: significant *p*-value (<0.05).

#### 1.2. ALDH2 and ADH1B genotyping

The ALDH2 active group, characterized by the presence of ALDH2*1/*1, comprised 35 patients, and the heterozygous inactive group, characterized by the presence of ALDH2*1/*2, comprised 17 patients. The ADH1B active group, characterized by the presence of ADH1B*1/*2 or ADH1B*2/*2, comprised 32 patients, and the less-active group, characterized by the presence of ADH1B*1/*1, comprised 20 patients. None of the alcoholic patients analyzed showed the ALDH2 homozygous inactive genotype encoded by ALDH2*2/*2.

The mean ages of the patients in the ALDH2-active, -inactive, ADH1B-active, and -less-active groups are shown in Table 1. There were no significant age differences among the groups.

#### 1.3. Smoking history

The patients were categorized according to their smoking history. Three of the patients had never smoked. Six patients were ex-smokers, who had given up smoking at least 5 years previously. Active smokers, including patients who had given up smoking within the last 5 years, were subcategorized into two groups – light smokers and heavy smokers – using the mean pack*year value for the patients as a whole, 44.2, as the benchmark. Light smokers were patients who had a smoking history of less that 45 pack*years, whereas heavy smokers had a smoking history of 45 pack*years or more. There were 21 light smokers and 22 heavy smokers.

The mean ages of the patients in the never-smoker, ex-smoker, light smoker, and heavy smoker groups are shown in Table 1. There were no significant age differences among the groups.

### 2. Telomere length analysis

A representative FISH image of an esophageal biopsy specimen is shown in Fig. 2b, and a summary of telomere length analysis is given in Table 1.

#### 2.1. DIUL size and NTCR

**Figure 3:**
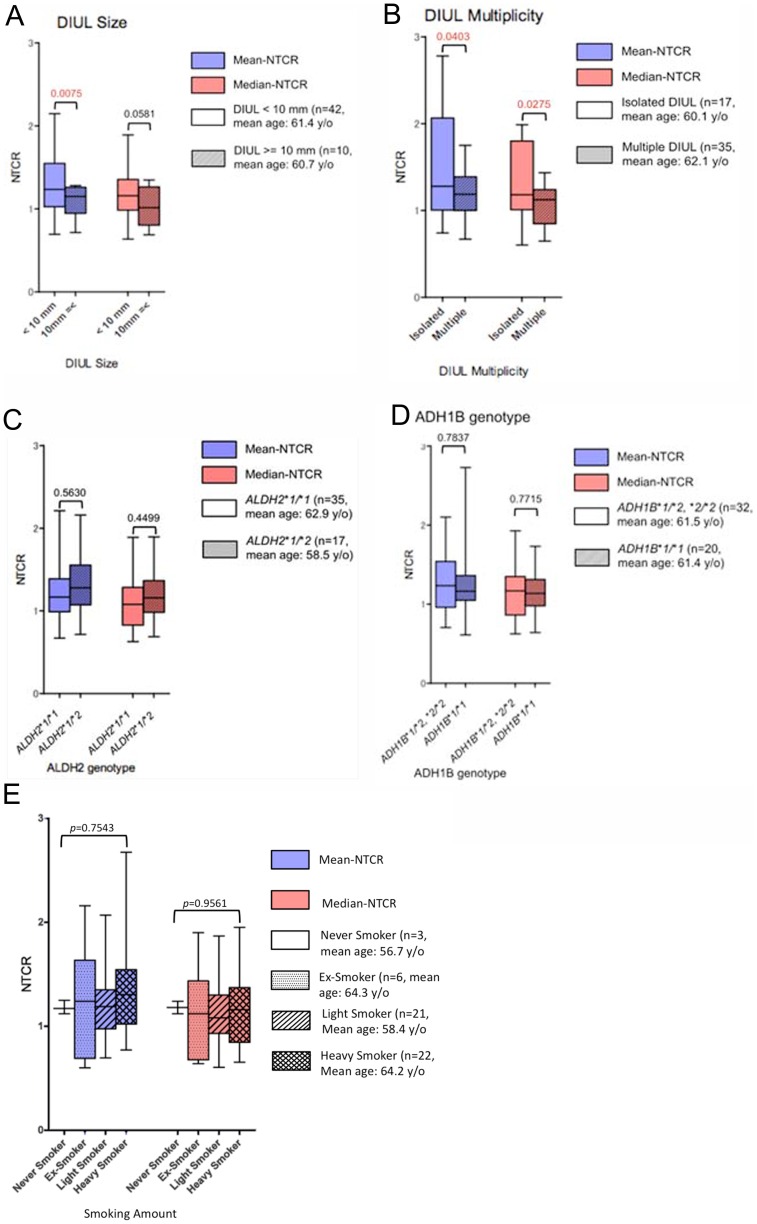
Comparison of NTCR among each group. Box and whisker plots of mean and median NTCRs of basal cells from DIULs in alcoholics, compared between groups for DIUL size (A), DIUL multiplicity (B), ALDH2 (C), ADH1B (D) and smoking history (E). *p*-values by *t* test (A–D) or Kruskal Wallis test (E) are shown in each graph. Differences at *p*-values of <0.05 were considered to be significant. Blue box; mean NTCR, red box; median NTCR. Clear box and shaded box indicate (A) DIUL <10 mm, and ≥10 mm (B) DIULs without, and with multiplicity (C) ALDH2*1/*1 and ALDH2*1/*2 (D) ADH1B*1/*2 or *2/*2, and ADH1B*1/*1. In Figure (E), the boxes indicate the following: clear box: never-smoker, dotted box: ex-smoker, shaded box: light smoker, and checked box: heavy smoker.

The mean NTCR in the group with larger DIULs was significantly higher than in the group with smaller DIULs (*p* = 0.0075). The median NTCR showed no significant inter-group difference (*p* = 0.0581) (Fig. 3A).

#### 2.2. Presence or absence of multiple DIULs and NTCR

Both the mean and median NTCRs were significantly smaller in the group with multiple DIULs than in the group without multiple DIULs (*p* = 0.0403, and 0.0275, respectively) (Fig. 3B).

#### 2.3. Combination analysis of size and multiplicity of DIULs and NTCR

For the combination of size and multiplicity (present or absent), we separated the cases into 4 groups (<10 mm and absent; SA, <10 mm and present; SP, ≥10 mm and absent; LA, ≥10 mm and present; LP), and compared the NTCR among them. Although no significant difference was found, the SA group had a larger NTCR than the SP group (Table S1).

#### 2.4. ALDH2 genotype and NTCR

There were no significant differences in mean and median NTCRs between the groups divided according to active or inactive ALDH2 genotype (*p* = 0.5630, 0.4499, respectively) (Fig. 3C).

#### 2.5. ADH1B genotype and NTCR

There were no significant differences in mean and median NTCRs between the groups divided according to active or less-active ADH1B genotype (*p* = 0.7837, 0.7715, respectively) (Fig. 3D).

#### 2.6. Combination analysis of ALDH2 and ADH1B genotype and NTCR

For the combination of ALDH2 and ADH1B genotype, we separated all the cases into 4 groups (ALDH2-active and ADH1B-active; AA, ALDH2-active and ADH1B-less-active; AL, ALDH2-inactive and ADH1B-active; IA, ALDH2-inactive and ADH1B-less-active), and compared among them. However, no significant differences in NTCR were evident among these 4 groups (Table S2).

#### 2.7. Smoking history

There were no significant differences in mean and median NTCRs among the groups categorized according to smoking history (*p* = 0.9561) (Fig. 3E).

### 3. Chi^-^square independence testing among the ALDH2 and ADH1B genotypes and size or multiplicity of DIULs

The ALDH2 genotype showed no association with the presence of DIULs ≥10 mm in diameter (chi-square  = 2.451, *p* = 0.117), but the ADH1B genotype did show such an association (chi-square  = 4.685, *p* = 0.030). Both the ALDH2 and ADH1B genotypes were also associated with the presence of multiple DIULs (chi-square  = 12.462, 8.683, *p* = 0.0004, 0.003, respectively).

## Discussion

The present study of alcoholics produced two main findings: (A) Telomeres were shorter in esophageal epithelium associated with DIULs ≥10 mm in diameter or multiple DIULs. (B) In DIULs of alcoholics, telomere lengths showed no significant differences between the ALDH2 and ADH1B genotypes, or between individuals with different smoking histories.

The International Agency for Research on Cancer of the World Health Organization has classified ethanol and acetaldehyde, which are associated with alcoholic beverages, as Group 1 human carcinogens [32]. In addition, ethanol is considered to be an inducer of P450 2E1 (CYP2E1), a co-carcinogen and/or tumor promoter [33]. In drinkers with the inactive ALDH2 genotype, the concentration of acetaldehyde markedly increases in blood and saliva, and probably in the esophageal mucosa. In drinkers with the less-active ADH1B genotype, oxidation of alcohol into acetaldehyde is delayed, leading to much longer exposure to alcohol after excessive alcohol intake. Multiple DIULs and large DIULs are strongly associated with the inactive ALDH2 and less-active ADH1B genotypes [20], [22], [23], and increase the risk of multifocal carcinogenesis, or “field carcinogenesis”, of the esophagus and head and neck in various Japanese populations [22], [23], [34]. Previously, we have reported that the background epithelium of the CIS has shorter telomeres and chromosomal instability than in controls [11], [17], and therefore we hypothesized that patients with inactive heterozygous ALDH2 genotypes and/or less-active homozygous ADH1B genotypes would have shorter telomeres. We considered that high levels of acetaldehyde accumulation and prolonged ethanol exposure in these individuals might to play an important role in accelerating telomere shortening.

The epithelial cells of DIULs ≥10 mm in diameter had shorter telomeres than those within smaller DIULs. When DIULs become larger, their epithelial telomeres might become shorter, and thus the epithelium in larger DIULs may have greater chromosomal instability than that in smaller DIULs, being compatible with the fact that SCC including CIS occurs more frequently in larger than in smaller DIULs [20]. When DIULs are increasing in size, their telomeres might be becoming shorter. In the combination of DIUL size or multiplicity, multiplicity is likely to have a close association with telomere shortening. Esophageal epithelium with multiple DIULs might generally contain shortened telomeres overall.

Pavanello and colleagues [35] have reported that although the telomere lengths of peripheral blood leukocytes in Italian alcohol abusers did not differ significantly among ADH1B genotypes, they were only about half those in normal controls; also, carriers of the less-active ADH1B genotype were more likely to be alcohol abusers, to have higher alcohol consumption, and to possess shorter telomeres. Analysis of the incidence of SCC between ALDH2 genotypes in Chinese and Japanese drinkers has shown that inactive heterozygous ALDH2 and less-active ADH1B are associated with an increased risk of SCC in both the esophagus and head and neck [34]. Although in the present study telomere shortening in the inactive ALDH2 and less-active ADH1B groups was expected, no significant difference in telomere length was evident between the active and less-active ALDH2 and ADH1B genotypes, or even their combinations. These results from alcoholics suggest two possibilities: 1) Irrespective of genotype, telomeres in the esophageal epithelium are shortened in alcoholics by heavy alcohol consumption; 2) DIULs are abnormal lesions in which telomere shortening has already occurred.

Alteration of p53 tumor suppressor protein is suspected to be the key molecular event in multifocal carcinogenesis in the esophagus, head and neck, and very high levels of p53 protein accumulation have been shown to occur in early esophageal SCC in Japanese alcoholic men [36]. Also, the expression of p53 protein in esophageal dysplasia has been found to correlate with the degree of atypia and to be positively associated with the size of DIULs and the presence of multiple DIULs [37]. Contrary to expectation, however, we did not recognize any correlation between the level of p53 protein accumulation in esophageal neoplasia and ALDH2/ADH1B genotypes in alcoholics [36], [37]. These findings are similar to the results of the present study regarding the telomere lengths of DIULs and ALDH2/ADH1B genotypes in alcoholics. Like p53 alterations, shortening of telomere length represents a very common molecular process in human neoplastic initiation and progression, and reflects the DIULs *per se* rather than the causative impact of ALDH2 and ADH1B genotype on the development of DIULs.

In any event, telomere shortening may not cause cancer directly, but may create conditions in which SCC can develop more easily, if subsequent exposure to carcinogens occurs.

## Conclusion

Our findings indicate that in DIULs of the esophagus in alcoholics, telomere lengths in epithelia do not differ significantly among individuals with different ALDH2 or ADH1B genotypes. Chromoendoscopy with iodine staining appears to provide evidence of telomere shortening in the esophageal epithelium.

## Supporting Information

Table S1 Combination of Diameter and Multiplicity of DIULs in NTCRs.
(DOCX)

Table S2 Combination of ALDH2 and ADH1B genotypes in NTCRs.
(DOCX)
