# Supplementary material for: Telomere Shortening in the Esophagus of Japanese Alcoholics: Relationships with Chromoendoscopic Findings, ALDH2 and ADH1B Genotypes and Smoking History
Source: PLoS One. 2013 May 7;8(5):e63860. doi: 10.1371/journal.pone.0063860 (PMC3646776; doi:10.1371/journal.pone.0063860)
Supplement: Table S1 — Combination of Diameter and Multiplicity of DIULs in NTCRs. (DOCX) [file pone.0063860.s001.docx]

| **Table S1. Combination of Diameter and Multiplicity of DIULs in NTCRs** | | | | | |
| --- | --- | --- | --- | --- | --- |
|  |  |  |  |  | |
|  | SA (n = 15) | SP (n = 27) | LA (n = 2) | LP (n = 8) | |
| SA |  | 0.03* | 0.49 | 0.07 | |
| SP | *0.04** |  | 1.00 | 0.36 | |
| LA | *0.38* | *0.64* |  | 0.67 | |
| LP | *0.07* | *0.25* | *0.86* |  | |
| SA: DIULs <10mm and without multiplicity | | | *: *p*<0.05 | | |
| SP: DIULs <10mm and with multiplicity | | |  | | |
| LA: 10mm< DIULs without multiplicity | | |  | |  |
| LP: and with multiplicity | | |  | |  |

Normal letters: *p*-value bye median-NTCR

*Italic letters*: *p*-value by mean-NTCR
