# Supplementary material for: Telomere Shortening in the Esophagus of Japanese Alcoholics: Relationships with Chromoendoscopic Findings, ALDH2 and ADH1B Genotypes and Smoking History
Source: PLoS One. 2013 May 7;8(5):e63860. doi: 10.1371/journal.pone.0063860 (PMC3646776; doi:10.1371/journal.pone.0063860)
Supplement: Table S2 — Combination of ALDH2 and ADH1B genotypes in NTCRs. (DOCX) [file pone.0063860.s002.docx]

| **Table S2. Combination of ALDH2 and ADH1B genotypes in NTCRs** | | | | | | | | |
| --- | --- | --- | --- | --- | --- | --- | --- | --- |
|  |  |  |  | | | |  | |
|  | AA (n = 22) | AL (n = 13) | IH (n = 10) | | | | IL (n = 7) | |
| AA |  | 0.84 | 0.61 | | | | 0.78 | |
| AL | *0.89* |  | 0.50 | | | | 0.60 | |
| IA | *0.40* | *0.59* |  | | | | 0.83 | |
| IL | *0.93* | *0.87* | *0.40* | | | |  | |
| AA: *ALDH2*1/*1* and *ADH1B*1/*2* or *ADH1B*2/*2*: | | |  | | | | | |
| AL: *ALDH2*1/*1* and *ADH1B*1/*1* | | |  | | | | | |
| IA: *ALDH2*1/*2* and *ADH1B*1/*2* or *ADH1B*2/*2* | | | |  | | | |  |
| IL: *ALDH2*1/*2* and *ADH1B*1/*1* | | | | |  |  | |  |

Normal letters: *p*-value bye median-NTCR

*Italic letters*: *p*-value by mean-NTCR
